# Implant allergy 

**DOI:** 10.5414/ALX01394E

**Published:** 2017-08-04

**Authors:** P. Thomas, B. Summer

**Affiliations:** Klinik und Poliklinik für Dermatologie und Allergologie, Ludwig-Maximilians-Universität, Munich, Germany

**Keywords:** osteosynthesis, endoprosthesis, implant, allergy, metal, nickel, chromium, cobalt, bone cement, patch test, histopathology

## Abstract

Osteosynthesis materials or artificial joint replacement make part of clinical routine. In case of complaints mostly mechanical causes or infections are found. Metals like nickel, chromium and cobalt or bone cement components like acrylates and gentamicine may however potentially cause intolerance reactions to implants. Correspondingly, eczema, delayed wound/bone healing, recurrent effusion, pain or implant loosening have been described as manifestation of implant allergy. In contrast to the high incidence of cutaneous metal allergy, allergies associated with implants are rare. Diagnosis of metal implant allergy is based on excluding differential diagnoses – in particular infection – and on a combined approach of allergological diagnostics by patch test and histopathology of periimplant tissue. Risk factors for allergic sensitization to implants or triggering periimplant allergic reactions in the case of preexisting cutaneous metal allergy are unknown. Despite the risk of developing complications being unclear, titanium-based osteosynthesis materials are recommended for metal-allergic patients and the use of metal-metal couplings in arthroplasty is rather not recommended for such patients. If a regular, potentially applicable CoCr-polyethylene articulation is preferred, the patient has to be well informed and has to give his written consent.

German version published in Allergologie, Vol. 34, No. 1/2011, pp. 42-48

## Introduction 

Osteosynthesis materials and artificial joint replacements – especially artificial hip and knee joints – are implanted more than 300,000 times per year in Germany alone. In case of complications mostly mechanical causes or infections are suspected [18]. 

Corrosion and abrasive particles lead to the release of metal. In this context nickel, chromium and cobalt as well as, occasionally, bone cement components have been described to induce implant allergy [38]. Increased rates of metal allergy (nickel, chromium and/or cobalt) have been reported in patients with old-generation (1975 – 1990) hip arthroplasty and metal-on-metal couplings, e.g., in metal-on-metal couplings of McKee-Farrar arthroplasty or in metal-on-plastic couplings (Charnley arthroplastic) [3]. A study published in 2005 [19] compared patients with hip athroplasty (ceramic, metal-on-plastic, metal-on-metal) of which 53 patients had stable and 104 had loosened hip replacements. The study demonstrated that allergies to metal or bone cement were not directly associated with implant failure, but with a worse 10-year implant survival rate (41.3% vs. 50.5%). Osteosynthesis using stainless steel can trigger eczemas and impede wound healing in nickel-allergic patients [4]. Of 239 patients with complaints due to metal replacements 29.7% had metal allergy: 21.3% of patients to nickel, 10.9% to cobalt and 5% to chromium (some patients were sensitized to more than one of these allergens) [13]. The relevance of contact allergies to metals or bone cement components as a potential cause of complications of endoprosthetic devices remains to be clarified. We report on a patient collective with complications of cemented knee/hip joint replacements in which a high rate of contact allergy to potential bone cement components, particularly gentamicine, was present [38]. For evaluation we initiated a control test study in complication-free endoprosthesis patients [12]. It is definitely possible that despite a cutaneous metal allergy, the same metals, when introduced into the body, are tolerated without any adverse reaction – as for example reported by Carlsson et al. [6, 7]. Neither Rooker and Wilkinson [34] nor Duchna et al. [11] found increased rates of metal allergy after implantation of metal replacements. Also studies by Rau and Thomson [31] as well as by Thyssen et al. [41] show that presumably only few patients with cutaneous contact allergy (to metals) develop complications after receiving metal endoprostheses. 

These controversial data show that a positive patch test to metal(s) is of only limited prognostic value for suspected periimplant hypersensitivity reactions [32]. Very few patients with endoprosthesis failure – in particularly when metal-on-metal replacements were used – do not show the usually detectable particle-associated foreign body reactions in the periimplant tissue, but lymphocytic infiltrates instead. The combination of lymphohistiocytic infiltrates, loosening of the implant, partial formation of effusions and the extensive lack of giant cell foreign body reaction was interpreted as local hypersensitivity reaction by Willert et al. [43, 44], Davies et al. [9] and Baur et al. [2]. Our own investigation on patients with revised metal-on-metal replacements and periimplant lymphocytic inflammation demonstrated a high coincidence with cutaneous contact allergy and metal-specific T-cell hyperreactivity in vitro [36]. 

## Implant material 

Mostly CoCr- and titanium alloys are used. The allergologically irrelevant polyethylene or ceramic materials will not be discussed here. For osteosynthesis steel-based and, more and more frequently, also titanium-based material is used. Bone cements are mostly acrylate-based. 

### CoCr alloys (mostly used as basic material for endoprosthetic devices) 

They mainly consist of cobalt. The composition (weight proportion) is usually ~ 64% cobalt, 28% chromium, 6% molybdenum and ~ 0.5% nickel [22, 40]. The nickel content can be as much as ~ 1%. If necessary, more specific details about the actually used alloy can provide further information on the proportions of substances released from the alloys. The weight proportion does not, however, reflect exactly the percentage of these metals released by corrosion or via abrasive particles. 

### Chromium-nickel steels (mostly used as basic material for osteosynthetic devices) 

The main component is iron. In addition, they contain ~ 18% chromium, up to 33% of nickel and ~ 3% molybdenum. Grade 316L stainless steel is only rarely used nowadays. They are more frequently used in steel wires (“Kirschner wires”, cerclage wires). 

### Titanium alloys 

The largest component is titanium (at least 87 wt%) with either 6% aluminum and 4% vanadium or 6% aluminum and 7% niobium. So-called b-titanium alloys have another composition. The “TMZF” alloy, for example, contains molybdenum (12%), zirconium (6%) and iron (2%). Acetabular cups and so-called pure titanium (> 99%) implant material is used. Minimal nickel impurities are possible and are between ~ 0.012 and 0.034 wt% [35]. A contamination with nickel during the manufacturing process and due to surgical instruments used for implantation might be possible. 

### Bone cements 

For the production of acrylate-based bone cements a methyl methacrylate-containing solution is mixed with a powdery component. The latter already contains polymerized poly(methyl methacrylate) (PMMA) “pellets”. To control the polymerization reaction the following additives are used: dibenzoyl peroxide, N,N-Dimethyl-p-toluidine or 2-[4-(Dimethylamino)phenyl]ethanol. Furthermore, contrast agents, stabilizers, dyes (like chlorophyllin-copper complex) and mostly also antibiotics like gentamicine are added. The composition varies depending on the manufacturer. 

### Modified implant materials for patients with metal allergies 

For patients with metal allergies there are implants [1] made of titanium alloys or bearing surfaces that have

- a 1- or 2-layer coating in order to reduce the release of metal ions,
- a multilayer coating in order to reduce the release of metal ions,
- an oxinium-based surface hardening/ceramization.

Long-term observational studies are necessary to evaluate the stability and effectiveness of the various alternative coating materials. This includes tests on the durability of the intact coating and on the potential danger of the spalling of coating particles that are very hard and can result in a shortened implant survival rate due to “third-body wear”. 

## Metal implant allergy – clinical pictures 

### Skin reactions 

**Figure 1.**
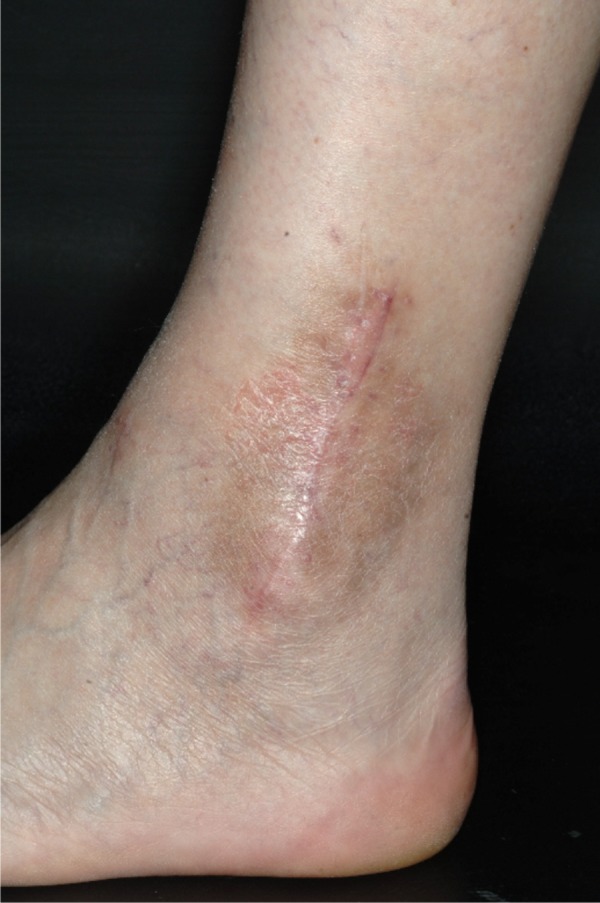
Eczema after osteosynthesis.

Skin symptoms in association with metal implants and metal allergy have been described as local eczemas, relapsing erysipelas-like livid redness and swelling as well as delayed wound healing [26]. Eczemas were mainly observed after (plate) osteosynthesis in the extremities in patients with nickel, chromium or cobalt allergy [4, 8, 14, 24]. Figure 1 shows a local eczema after osteosynthesis of an ankle fracture in a patient with nickel allergy. In patients with sternotomy and nickel allergy eczemas were reported even after steel wire cerclage [21]. The persistent redness, pruritus and big toe swelling in a nickel-allergic patient receiving corrective osteotomy with Kirschner wires is another example for osteosynthesis-associated skin reaction [16]. There are also case reports on metal-allergic patients developing corrosion-dependent eczemas over metal fragments that remained close to the skin [37]. In rare cases eczemas can also occur over artificial knees and hips [30, 39]. Skin hemorrhages in the form of vasculitis or urticaria are even rarer [29]. There are also reports on fistula formation due to intolerance to bone cement [33, 42]. 

### Further clinical manifestations 

Further clinical manifestations include impaired wound and fracture healing as well as, particularly in knee replacements, relapsing pain, loss of motion and effusions without prove for infection but with co-existing metal allergy [13]. Also in the case of hip replacements – especially for metal-on-metal couplings – relapsing pain and/or implant loosening without other causes have been described. In the synopsis of metal allergy and lymphocytic inflammation such cases were interpreted as metal implant allergy [9, 20, 36, 44]. Under some circumstances the involvement of allergy-related symptoms is being discussed: some cases with aseptic implant loosening and osteolysis around the implant [27]; patients developing persistent groin/hip pain and cystic pseudotumors after arthroplasty with metal-on-metal couplings [28]; patients with loss of motion in the replaced joint with the clinical picture of arthrofibrosis [25]. 

### Diagnostic work-up when implant intolerance is suspected 

**Figure 2.**
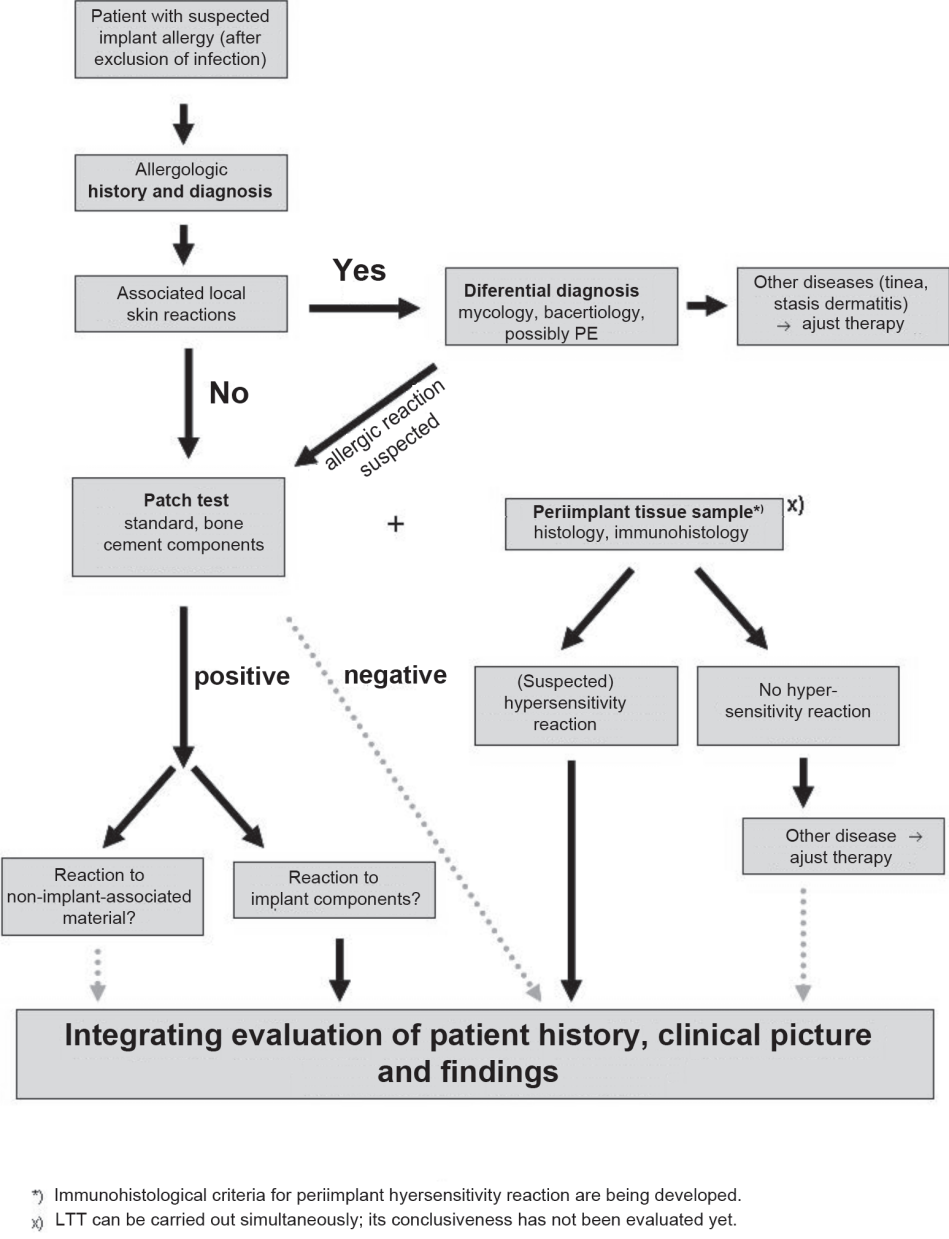
Flow chart for diagnostic work-up (modified according to Thomas and Thomsen, 2008).

Figure 2 shows a possible algorithm for diagnostic work-up. Before the allergologic diagnostic work-up orthopedic-surgical examinations have to be carried out to exclude differential diagnoses – particularly (low-grade) infection [39]. Reports on earlier complications associated with metal implants that were suspected to be allergy-related or an intolerance to acrylate-based materials, like dental plastic, can provide useful information for the allergologic history. Furthermore, in the case of “eczemas” near the implant other allergy sources (disinfectants, skin care products) and potential cutaneous conditions (tinea) have to be excluded. It is always necessary to scrutinize positive patch test results regarding their clinical relevance. The histopathology of the periimplant tissue can be an additional diagnostic step. The lymphocyte transformation test (LTT) can indicate sensitization to metals (which has mainly been shown for nickel [15]), but does not allow conclusions regarding a pathogenic hypersensitivity. 

### Patch testing 

Nickel, chromium or cobalt test preparations are included in the standard test series. For the diagnosis of contact allergy to other alloy metals no sufficiently tested preparations are available. We do not recommend testing with “alloy platelets”. 

For the testing with bone cement components acrylates and additives like gentamicine, for which also periimplant exposure of the patient is possible, can be used. In order to assess the reaction to gentamicine we also carry out late-readings after 7 days because late-type reactions have been observed several times. 

In their comment on allergologic diagnosis in cases of suspected implant intolerance the Deutsche Kontaktallergiegesellschaft (German Contact Allergy Society) pointed out [17] that the diagnostic work-up in these cases is an object of allergologic research, and as such far from any standardization. 

### Histology 

Periimplant tissue should be fixed in formalin and further (immuno)histological examinations regarding inflammatory cell infiltration (in particular T-cell-mediated inflammation), foreign body reaction or infection-related changes should be carried out. For cases of implant loosening there is a consensus classification with four histopathological patterns [25]: in Type I (abrasive type) the infiltration consists of macrophages and multinucleated giant cells; in Type II (infectious type) a pronounced or minimal infection with chronic granulomatous inflammation can be present; Type III (mixed type) is a combination of Type I and Type II; in Type IV there are only few cells and many collagen fibers. Late-type hypersensitivity is being discussed in the context of lymphocytic infiltration patterns: diffuse pattern, perivascular pattern and follicle-like structures have been described [45]. For a subtype Willert suggested the term Aseptic Lymphocytic Vasculitis-Associated Lesion (ALVAL) in 2001. 

## Summary 

Diagnosis of metal implant allergy should always be made in consideration of clinical findings (especially after orthopedic differential diagnoses have been excluded), results of patch testing and periimplant histopathology. This means that only in the synopsis of several diagnostic steps and findings the symptoms can be interpreted as allergic reaction and the patch test result is accordingly only partially applicable to periimplant tissue. At least, there is increasing interest in “implant allergy” and cases have been reported in which the patients were symptom-free after the diagnosis of metal implant allergy had been made and alternative materials had been used [10, 23]. Interdisciplinary cooperation will be necessary in order to be able to re-formulate the recommendations published in 2008, and co-authored by P. Thomas [39], more precisely on the basis of better data. 

## References

[1] Bader R Bergschmidt P Fritsche A Ansorge S Thomas P Mittelmeier W Alternativmaterialien für Knieendoprothetik bei Patienten mit Metallallergie. Orthopade. 2008; 37: 136–142. 18210089 10.1007/s00132-007-1189-x

[2] Baur W Hönle W Willert HG Schuh A Pathologische Veränderungen im umgebenden Gewebe von revidierten Metall-Metall-Gleitpaarungen. Orthopade. 2005; 34: 225–226, 228-233.. 15666136 10.1007/s00132-004-0761-x

[3] Benson MK Goodwin PG Brostoff J Metal sensitivity in patients with joint replacement arthroplasties. BMJ. 1975; 4: 374–375. 1192078 10.1136/bmj.4.5993.374 PMC1675237

[4] Brehler R Grabbe J Eichelberg D Nickelallergie nach Plattenosteosynthese. Akt Dermatol.. 1990; 16: 202–203.

[5] Breusch SJ Kühn KD Bone cements based on polymethylmethacrylate. Orthopade. 2003; 32: 41–50. 12557085 10.1007/s00132-002-0411-0

[6] Carlsson A Möller H Implantation of orthopaedic devices in patients with metal allergy. Acta Derm Venereol. 1989; 69: 62–66. 2563611

[7] Carlsson AS Magnusson B Möller H Metal sensitivity in patients with metal-to-plastic total hip arthroplasties. Acta Orthop Scand. 1980; 51: 57–62. 7376846 10.3109/17453678008990769

[8] Cramers M Lucht U Metal sensitivity in patients treated for tibial fractures with plates of stainless steel. Acta Orthop Scand. 1977; 48: 245–249. 920115 10.3109/17453677708988763

[9] Davies AP Willert HG Campbell PA Learmonth ID Case CP An unusual lymphocytic perivascular infiltration in tissues around contemporary metal-on-metal joint replacements. J Bone Joint Surg Am. 2005; 87: 18–27. 15634811 10.2106/JBJS.C.00949

[10] Dietrich KA Mazoochian F Summer B Reinert M Ruzicka T Thomas P Intolerance reactions to knee arthroplasty in patients with nickel/cobalt allergy and disappearance of symptoms after revision surgery with titanium-based endoprostheses. J Dtsch Dermatol Ges. 2009; 7: 410–413. 19192161 10.1111/j.1610-0387.2008.06987.x

[11] Duchna HW Nowack U Merget R Muhr G Schultze-Werninghaus G Prospektive Untersuchung zur Bedeutung der Kontaktsensibilisierung durch Metallimplantate. Zentralbl Chir. 1998; 123: 1271–1276. 9880846

[12] Eben R Dietrich KA Nerz C Schneider S Schuh A Banke IJ Mazoochian F Thomas P Contact allergy to metals and bone cement components in patients with intolerance of arthroplasty. Dtsch Med Wochenschr. 2010; 135: 1418–1422. 20614402 10.1055/s-0030-1262426

[13] Eben R Walk R Summer B Maier S Thomsen M Thomas P Implantatallergieregister – ein erster Erfahrungsbericht. Orthopade. 2009; 38: 557–562. 19468709 10.1007/s00132-009-1414-x

[14] Ebert B Metallallergisches Ekzem nach Osteosynthese. Akt Dermatol.. 1993; 19: 9–12.

[15] Eis D Wolf U alitätssicherung beim Lymphozytentransformationstest“-Addendum zum LTT-Papier der RKI-Kommission „Methoden und Qualitätssicherung in der Umweltmedizin“. Mitteilung der Kommission „Methoden und Qualitätssicherung in der Umweltmedizin“. Bundesgesundheitsblatt Gesundheitsforschung Gesundheitsschutz. 2008; 51: 1070–1076. 18773166 10.1007/s00103-008-0641-3

[16] Gabel M Summer B Thomas P Persistierende Entzündung nach Grosszehenkorrekturoperation bei einer Patientin mit Nickelallergie: Manifestation einer Überempfindlichkeit gegen Metallpartikel? Fuss Sprunggelenk. 2008; 6: 160–165.

[17] Geier J Lessmann H Becker D Thomas P Allergologische Diagnostik bei Verdacht auf Implantatunverträglichkeit: Hinweise für die Praxis. Eine Stellungnahme der Deutschen Kontaktallergie-Gruppe (DKG). Hautarzt. 2008; 59: 594–597. 18542895 10.1007/s00105-008-1587-y

[18] Gollwitzer H Diehl P Gerdesmeyer L Mittelmeier W Diagnostic strategies in cases of suspected periprosthetic infection of the knee. A review of the literature and current recommendations. Orthopade. 2006; 35: 904–916, 906-908, 910-916.. 16794850 10.1007/s00132-006-0977-z

[19] Granchi D Cenni E Trisolino G Giunti A Baldini N Sensitivity to implant materials in patients undergoing total hip replacement. J Biomed Mater Res B Appl Biomater. 2006; 77: 257–264. 16265661 10.1002/jbm.b.30445

[20] Hallab NJ Jacobs JJ Biologic effects of implant debris. Bull NYU Hosp Jt Dis. 2009; 67: 182–188. 19583551

[21] Hayashi K Kaneko H Kawachi S Saida T Allergic contact dermatitis and osteomyelitis due to sternal stainless steel wire. Contact Dermat. 1999; 41: 115–116. 10.1111/j.1600-0536.1999.tb06249.x 10445705

[22] Holzwarth U Thomas P Kachler W Goske J Schuh A Metallkundliche Differenzierung heutiger Kobaltbasis-Implantatlegierungen. Orthopade. 2005; 34: 1046–1051. 16091961 10.1007/s00132-005-0849-y

[23] Jensen P Thyssen JP Retpen JB Menné T Cobalt allergy and suspected aseptic lymphocyte-dominated vascular-associated lesion following total hip arthroplasty. Contact Dermat. 2009; 61: 238–239. 10.1111/j.1600-0536.2009.01599.x 19825098

[24] Kanerva L Förström L Allergic nickel and chromate hand dermatitis induced by orthopaedic metal implant. Contact Dermat. 2001; 44: 103–104. 10.1034/j.1600-0536.2001.4402096.x 11205382

[25] Krenn V Otto M Morawietz L Hopf T Jakobs M Klauser W Schwantes B Gehrke T Histopathologische Diagnostik in der Endoprothetik: Periprothetische Neosynovialitis, Hypersensitivitätsreaktion und Arthrofibrose. Orthopade. 2009; 38: 520–530. 19448983 10.1007/s00132-008-1400-8

[26] Kubba R Taylor JS Marks KE Cutaneous complications of orthopedic implants. A two-year prospective study. Arch Dermatol. 1981; 117: 554–560. 7294846

[27] Looney RJ Schwarz EM Boyd A O’Keefe RJ Periprosthetic osteolysis: an immunologist’s update. Curr Opin Rheumatol. 2006; 18: 80–87. 16344623 10.1097/01.bor.0000198004.88568.96

[28] Mahendra G Pandit H Kliskey K Murray D Gill HS Athanasou N Necrotic and inflammatory changes in metal-on-metal resurfacing hip arthroplasties. Acta Orthop. 2009; 80: 653–659. 19995315 10.3109/17453670903473016 PMC2823316

[29] McKenzie AW Aitken CV Ridsdill-Smith R Urticaria after insertion of Smith-Petersen Vitallium nail. BMJ. 1967; 4: 36. 6047831 10.1136/bmj.4.5570.36 PMC1748860

[30] Merritt K Brown SA Distribution of cobalt chromium wear and corrosion products and biologic reactions. Clin Orthop Relat Res. 1996; 329: S233–S243. 10.1097/00003086-199608001-00020 8769337

[31] Rau C Thomas P Thomsen M Metallallergie bei Patienten vor bzw. nach endoprothetischem Gelenkersatz. Orthopade. 2008; 37: 102–110. 18210091 10.1007/s00132-007-1186-0

[32] Reed KB Davis MD Nakamura K Hanson L Richardson DM Retrospective evaluation of patch testing before or after metal device implantation. Arch Dermatol. 2008; 144: 999–1007. 18711071 10.1001/archderm.144.8.999

[33] Richter-Hintz D Rieker J Rauch L Homey B Prothesenunverträglichkeit bei Typ-IV-Sensibilisierung gegen Knochenzement. Hautarzt. 2004; 55: 987–989. 15351871 10.1007/s00105-004-0814-4

[34] Rooker GD Wilkinson JD Metal sensitivity in patients undergoing hip replacement. A prospective study. J Bone Joint Surg Br. 1980; 62-B: 502–505. 7430234 10.1302/0301-620X.62B4.7430234

[35] Schuh A Thomas P Kachler W Göske J Wagner L Holzwarth U Forst R Das Allergiepotenzial von Implantatwerkstoffen auf Titanbasis. Orthopade. 2005; 34: 327–328 , 330-333.. 15706453 10.1007/s00132-005-0764-2

[36] Thomas P Braathen LR Dörig M Auböck J Nestle F Werfel T Willert HG Increased metal allergy in patients with failed metal-on-metal hip arthroplasty and peri-implant T-lymphocytic inflammation. Allergy. 2009; 64: 1157–1165. 19220218 10.1111/j.1398-9995.2009.01966.x

[37] Thomas P Gollwitzer H Maier S Rueff F Osteosynthesis associated contact dermatitis with unusual perpetuation of hyperreactivity in a nickel allergic patient. Contact Dermat. 2006; 54: 222–225. 10.1111/j.0105-1873.2006.0775j.x 16650106

[38] Thomas P Schuh A Eben R Thomsen M Allergie auf Knochenzementbestandteile. Orthopade. 2008; 37: 117–120. 18227996 10.1007/s00132-008-1195-7

[39] Thomas P Schuh A Ring J Thomsen M Orthopädisch-chirurgische Implantate und Allergien: Gemeinsame Stellungnahme des Arbeitskreises Implantatallergie (AK 20) der Deutschen Gesellschaft für Orthopädie und Orthopädische Chirurgie (DGOOC), der Deutschen Kontaktallergie Gruppe (DKG) und der Deutschen Gesellschaft für Allergologie und Klinische Immunologie (DGAKI). Orthopade. 2008; 37: 75–88. 18210082 10.1007/s00132-007-1183-3

[40] Thomsen M von Strachwitz B Mau H Cotta H Werkstoffübersicht in der Hüftendoprothetik. Z Orthop Ihre Grenzgeb. 1995; 133: 1–6. 10.1055/s-2008-1039450 7886993

[41] Thyssen JP Jakobsen SS Engkilde K Johansen JD Søballe K Menné T The association between metal allergy, total hip arthroplasty, and revision. Acta Orthop. 2009; 80: 646–652. 19995314 10.3109/17453670903487008 PMC2823320

[42] Wetzel S Thomas P Allergie gegen Implantatwerkstoffe In: Plewig G, Kaudewitz P, Sander C. Fortschritte der praktischen Dermatologie und Venerologie. Berlin: Springer; 2004 p. 817-818.

[43] Willert HG Buchhorn A Fayyazi A Lohmann CH Histopathologische Veränderungen bei Metall/Metall-Gelenken geben Hinweise auf eine zellvermittelte Überempfindlichkeit. Osteologie. 2000; 9: 165–179.

[44] Willert HG Buchhorn GH Fayyazi A Flury R Windler M Köster G Lohmann CH Metal-on-metal bearings and hypersensitivity in patients with artificial hip joints. A clinical and histomorphological study. J Bone Joint Surg Am. 2005; 87: 28–36. 10.2106/JBJS.A.02039pp 15637030

[45] Witzleb WC Hanisch U Kolar N Krummenauer F Guenther KP Neo-capsule tissue reactions in metal-on-metal hip arthroplasty. Acta Orthop. 2007; 78: 211–220. 17464609 10.1080/17453670710013708

